# Sprouty2 Suppresses Epithelial-Mesenchymal Transition of Human Lens Epithelial Cells through Blockade of Smad2 and ERK1/2 Pathways

**DOI:** 10.1371/journal.pone.0159275

**Published:** 2016-07-14

**Authors:** Xuhua Tan, Yi Zhu, Chuan Chen, Xiaoyun Chen, Yingyan Qin, Bo Qu, Lixia Luo, Haotian Lin, Mingxing Wu, Weirong Chen, Yizhi Liu

**Affiliations:** State Key Laboratory of Ophthalmology, Zhongshan Ophthalmic Center, Sun Yat-sen University, Guangzhou, Guangdong, China; University of South Alabama Mitchell Cancer Institute, UNITED STATES

## Abstract

Transforming growth factor β (TGFβ)-induced epithelial-mesenchymal transition (EMT) of lens epithelial cells (LECs) plays a key role in the pathogenesis of anterior subcapsular cataract (ASC) and capsule opacification. In mouse lens, Sprouty2 (Spry2) has a negative regulatory role on TGFβ signaling. However, the regulation of Spry2 during ASC development and how Spry2 modulates TGFβ signaling pathway in human LECs have not been characterized. Here, we demonstrate that Spry2 expression level is decreased in anterior capsule LECs of ASC patients. Spry2 negatively regulates TGFβ2-induced EMT and migration of LECs through inhibition of Smad2 and ERK1/2 phosphorylation. Also, blockade of Smad2 or ERK1/2 activation suppresses EMT caused by Spry2 downregulation. Collectively, our results for the first time show in human LECs that Spry2 has an inhibitory role in TGFβ signaling pathway. Our findings in human lens tissue and epithelial cells suggest that Spry2 may become a novel therapeutic target for the prevention and treatment of ASC and capsule opacification.

## Introduction

Anterior subcapsular cataract (ASC) and capsule opacification are both caused by excessive proliferation and differentiation of lens epithelial cells (LECs)[1–4]. ASC is a primary cataract characterized by star-shaped or irregular fibrotic plaques beneath the anterior capsule, resulting in dramatic visual reduction due to visual axis involvement[5]. Capsule opacification is one of the most common complications after cataract surgery. Posterior capsule opacification (PCO), also known as secondary cataract, results from proliferation and migration of residual lens epithelial cells across the posterior capsule. About 20%-40% adult patients develop PCO within 5 years after surgery, and the incidence is almost 100% in children[6–8]. On the other hand, anterior capsule opacification (ACO) occurs around capsulotomy edge and usually develops faster than PCO. Excessive ACO leads to capsule shrinkage, IOL decentration, capsule contraction syndrome and limits peripheral fundus examination[9, 10].

Proliferation and epithelial-mesenchymal transition (EMT) of LECs play key roles in the pathogenesis of ASC and capsule opacification[4, 11, 12]. During EMT, LECs undergo cytoskeletal rearrangement, lose epithelial polarity, and transdifferentiate into active fibroblast-like cells[13]. EMT is also a crucial pathologic change in various fibrotic diseases and cancer metastasis[14, 15]. Transforming growth factor β (TGFβ) is the most potent inducer of EMT[16]. Canonical TGFβ signaling requires phosphorylation of Smad2 and Smad3, which then translocate into nucleus and turn on the expression of target genes, such as α-SMA, fibronectin (Fn), vimentin (Vim), collagen I (Col I), and collagen IV (Col IV)[17]. Also, TGFβ can activate extracellular signal-regulated kinase 1/2 (ERK1/2), p38 MAPK, JNK, Rho-like GTPase and Jagged/Notch as non-canonical pathways to induce EMT[18–21]. There are three isoforms of TGFβ (TGFβ1–3) in mammals[22]. TGFβ2 is the major form in aqueous humor, and is significantly upregulated after injury or during inflammation[23–25]. Therefore, inhibition of TGFβ2-induced EMT is considered to be a promising therapeutic strategy for ASC and capsule opacification[5, 26].

Sprouty (Spry) protein family is a highly conserved group of inhibitors that suppress ERK1/2 activation in various receptor tyrosine kinase (RTK) pathways[27, 28]. It was first reported in *Drosophila* as an antagonistic regulator of fibroblast growth factor (FGF) and epidermal growth factor(EGF) signaling[29]. Spry is widely considered as a tumor suppressor, and downregulation of Spry has been found in prostate, breast, liver and lung cancer, especially in the metastatic stages[30]. Also, overexpression of Spry can inhibit tumorigenesis[31]. To date, four mammalian Spry members (Spry1-4) have been identified. Of them, Spry2 is the major isoform expressed in mice mature lens fiber cells[32]. During lens development, Spry2 negatively modulates ERKs to allow lens vesicle separation[33]. Conditional knockout of Spry2 in mouse lens enhances TGFβ-induced EMT, while Spry2 overexpression inhibits LEC proliferation and differentiation[34–36]. These studies highlight the importance of Spry2 in lens development and cataractogenesis in mouse. However, the role of Spry2 in human ASC and capsule opacification formation has not been studied, and the molecular mechanism of Spry2-regulated TGFβ signaling in human lens is still largely unknown.

Here we seek to address the regulatory role of Spry2 on TGFβ-induced EMT in human LECs. We compared the RNA and protein levels of Spry2 in anterior capsule LECs from ASC patients with those from age-matched controls, and measured EMT level upon Spry2 downregulation or overexpression in human LECs. Our results demonstrate that Spry2 suppresses EMT of LECs by inhibiting both the canonical Smad pathway and the non-canonical ERK1/2 pathway, suggesting that Spry2 may be potentially a potent target for modulation of TGFβ-induced EMT in human LECs.

## Materials and Methods

### Human anterior capsule samples collection

Anterior capsule specimens with LECs from ASC and age-matched cortical cataract patients were obtained during cataract surgery. Each capsule is about 5 mm in diameter and contains the central area. Written informed consent forms were obtained from the patients before surgery, and the tenets of the Declaration of Helsinki were followed throughout the study. In addition, age-matched postmortem anterior capsule specimens of transparent lens obtained within 6 hours from death were used as controls. Cadaver eye tissues were obtained from the eye bank of Zhongshan Ophthalmic Center. The research protocol was approved by the Institutional Review Board/Ethics Committee of the Sun Yat-sen University.

### Cell culture

Human lens epithelial cell line SRA01/04 was kindly provided by Professor Fu Shang at the Laboratory for Nutrition and Vision Research (Boston, MA, USA), and cultured in Dulbecco's modified Eagle's medium (DMEM) containing 10% fetal bovine serum (FBS). Cells were grown in a humidified 37°C incubator with 5% CO_2_ and dissociated with 0.25% trypsin-0.02% ethylenediamine-tetraacetic acid (EDTA). For TGFβ2 treatment, cells were seeded in six-well plates with the density of 1×10^5^/well, incubated in serum-free medium for 5 hours, and treated with 10 ng/mL TGFβ2 (Cell Signaling Technology, Danvers, MA, USA).

### siRNA and plasmid transfection

To knockdown (KD) Spry2, Spry2 siRNA (sc-41037, Santa Cruz Biotechnology, CA, USA) or scrambled siRNA (sc-37007, Santa Cruz Biotechnology) were transfected into SRA01/04 cells using Lipofectamine 2000 (Invitrogen, CA, USA) according to the manufacturer’s protocol. Cells transfected with various concentrations of FITC-conjugated siRNA (sc-36869, Santa Cruz Biotechnology) were subjected to flow cytometry to monitor transfection efficiency, and an optimal siRNA concentration of 80nM was determined. To overexpress Spry2, human Spry2 cDNA plasmid (RC204864, Origene, Rockville, MD, USA) was transfected into SRA01/04 cells using X-tremeGENE HP DNA transfection reagent (Roche, Basel, Switzerland) according to the manufacturer’s protocol. Transfection of LECs with empty pCMV6-Entry vector (PS100001, Origene) was used as a negative control.

### Western blot

For total protein extraction, cells were lysed in RIPA buffer with protease inhibitor cocktail. After mixing with 5× SDS sample buffer, protein samples were separated by 10% sodium dodecyl sulfate-polyacrylamide gel electrophoresis (SDS-PAGE) and transferred to polyvinyl difluoride (PVDF) membranes (Bio-Rad). The membrane was blocked in 5% nonfat milk and incubated with primary antibody at 4°C overnight, and washed with PBST (0.1% Tween-20 in PBS). Then the membrane was incubated with horseradish peroxidase (HRP)-conjugated secondary antibodies for 1 hour at room temperature, and washed with PBST. Protein bands were detected with chemiluminescence detection reagents. β-actin was used as a loading control. Quantitative analysis was done using Image J 1.41 (National Institutes of Health, Bethesda, MD, USA). The sources and dilutions of antibodies are: rabbit anti-Spry2 (1:200, Santa Cruz Biotechnology), rabbit anti-ERK (1:1000, Cell Signaling Technology), rabbit anti-p-ERK (1:1000, Thr 202/Tyr 204, Cell Signaling Technology), rabbit anti-Smad2 (1:1000, Cell Signaling Technology), rabbit anti-p-Smad2 (1:1000, Ser 465/467, Cell Signaling Technology), mouse anti-α-SMA (1:200, Abcam, MA, USA), rabbit anti-fibronectin (1:200, Abcam), rabbit anti-collagen type I (1:1000, Abcam), rabbit anti-collagen type IV (1:500, Abcam), rabbit anti-actin (1:3000, Abcam), horseradish peroxidase (HRP)-conjugated horse anti-mouse IgG (1:2000, Cell Signaling Technology) and HRP-conjugated goat anti-rabbit IgG (1:2000, Cell Signaling Technology).

### RNA extraction and quantitative PCR

Total RNA was extracted from fresh anterior capsule tissue or LECs using Trizol reagent (Invitrogen, Carlsbad, CA, USA) according to the manufacturer’s protocol. RNA concentration was measured spectrophotometrically at 260nm. 2μg RNA was used for reverse transcription to cDNA using a reverse transcription kit (Takara, Siga, Japan). SYBR PrimeScript RT-PCR kit (Takara) was used to amplify target genes by the ABI Prism 7000 sequence detection system (Applied Biosystems, Foster City, CA, USA). Glyceraldehyde 3-phosphate dehydrogenase (GAPDH) was used as an internal control. Primer sequences were listed in Table 1. All the primers were synthesized by Beijing Genomics Institute (Beijing, China).

**Table 1:** Primers used for real-time quantitative PCR.

Gene	Forward primer	Reverse primer
**Spry2**	5'-ATCCAGAGACAAGACATGTAC-3'	5'-TTCAGATGTGTTCTAAGCC-3'
**α-SMA**	5'-CCGACCGAATGCAGAAGGA-3'	5'-ACAGAGTATTTGCGC-TCCGAA-3'
**Fibronectin**	5'-GAGCTGCACATGTCTTGGGAAC-3'	5'-GGAGCAAATGGCACCGAGATA-3'
**Vimentin**	5'-TGAGTACCGGAGACAGGTGCAG-3'	5'-TAGCAGCTTCAACGGCAAAGTTC-3'
**GAPDH**	5'-GAGTCAACGGATTTG-GTCGT-3'	5'-AATGAAGGGGTCATTGATGG-3'

### Immunofluorescence Staining

For cell staining, SRA 01/04 cells were seeded on Millicell EZ 4-well glass slides (Millipore, MA, USA). After treatment for indicated times, cells were fixed with 4% paraformaldehyde, permeabilized with 0.1% Triton X-100 and blocked with 1% bovine serum albumin (BSA). Cells were then incubated with primary antibodies at 4°C overnight, and incubated with secondary antibody for 1 hour at room temperature. The sources and dilutions of antibodies are: rabbit anti-Spry2 (1:50, Santa Cruz Biotechnology), rabbit anti-fibronectin (1:100, Abcam), rabbit anti-collagen type I (1:500, Abcam), rabbit anti-collagen type IV (1:200, Abcam), Alexa Fluor 488-conjugated goat anti-mouse IgG (1:1000, Cell Signaling Technology) and Alexa Fluor 555-conjugated donkey anti-rabbit IgG (1:1000, Cell Signaling Technology). Cell nuclei were stained with 50 ng/ml 4’,6-diamidino-2-phenylindole (DAPI) for 5 min. Slides were mounted with anti-fade fluorescent mounting medium (Applygen, #C1210). Images were acquired by a Zeiss LSM 510 confocal laser scanning microscope (CLSM, Carl Zeiss, Germany) and processed by Adobe Photoshop CS6.

For whole mount staining, anterior capsules were acquired from the capsulorhexis during cataract surgery. Immunofluorescence staining was performed as described above. Finally, the whole anterior capsule was placed flat on a microscope slide, and the capsule was covered with a coverslip after adding a drop of anti-fade mounting medium. The central region of each anterior capsule was scanned by a CLSM.

### Scratch wound assay

Scratched wound assay was used to evaluate the effect of Spry2 on TGFβ2-induced migration of LECs. Briefly, cells were seeded on a 6-well plate with the concentration of 1.5 x 10^5^/well, transfected with Spry2 siRNA or plasmid, and grown for 36 hours to reach 90% confluence. Cell monolayer was wounded by a 200-μl micropipette tip and washed with PBS for four times with to remove cell debris. Then the cells were incubated in 0.5% FBS medium with or without 10 ng/mL TGFβ2 for 36 hours. Migration of cells into the wound was examined by inverted microscope and photographed in a digital format (×100). Cell number was counted using Image-Pro Plus software 5.1 (Media Cybernetics, Inc. Silver Spring, MD, USA). In each group, six randomly chosen microscopic fields were analyzed, and the average cell number was calculated.

### Statistical analysis

Experiments presented in the figures are representative of at least three repetitions. All data are represented as means ± S.E.M. SPSS 17.0 software package (SPSS Inc., Chicago, IL, USA) was used for statistical analysis. One-way analysis of variance (ANOVA) followed by Bonferroni's post hoc test was used to compare means among three or more groups. Independent samples *t*-test was used to compare means between two groups. All statistical tests were two tailed. Values of P <0.05 were considered statistically significant.

## Results

### Expression level of Spry2 is downregulated in the anterior capsule LECs of ASC patients

**Fig 1:**
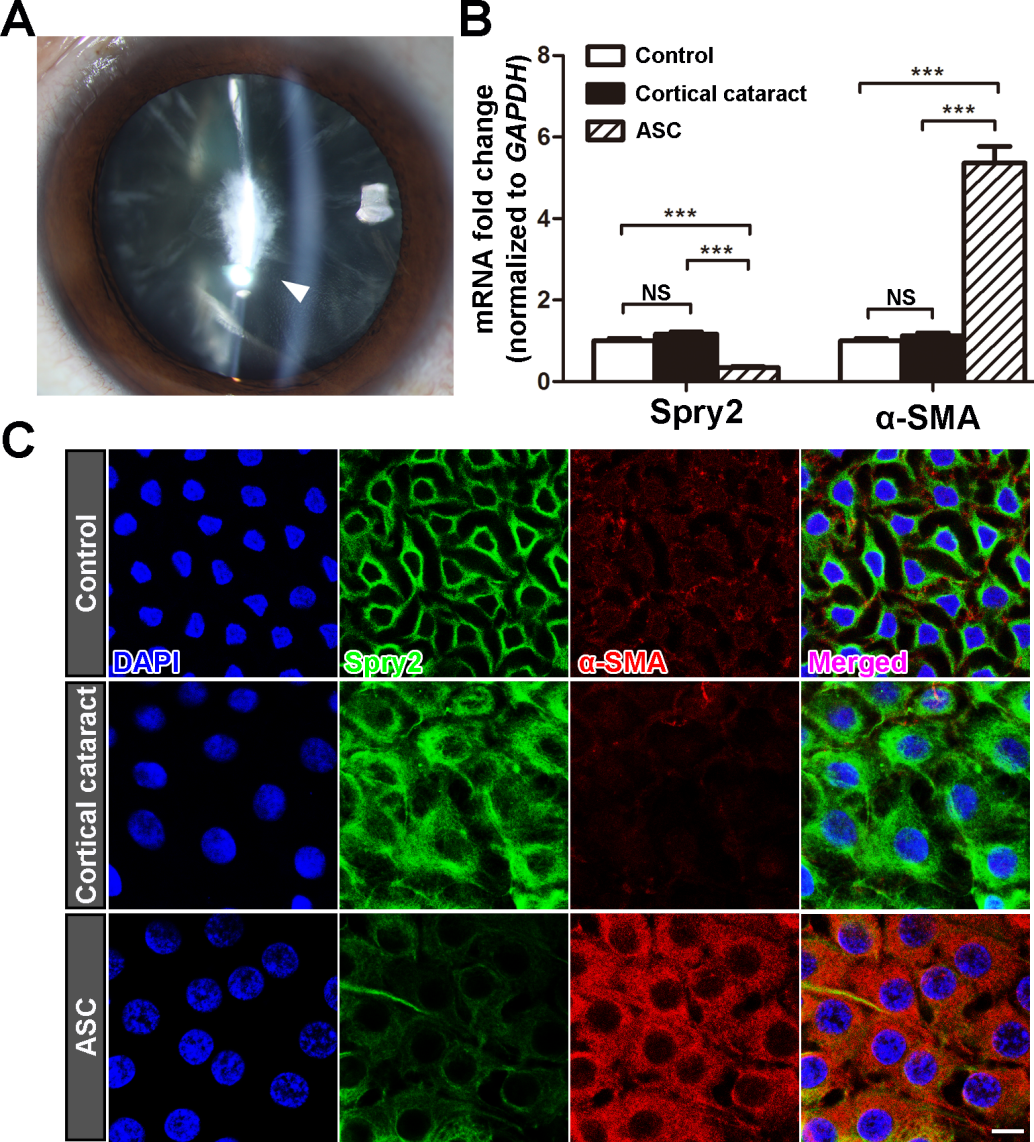
Spry2 expression level is decreased in anterior capsule LECs of ASC patients. (**A**) Representative slit-lamp microscope photo of an ASC patient (60 years old, Female). The white arrow indicates the irregular fibrotic opacity beneath the anterior capsule. (**B**) Total RNA was extracted from the anterior capsules of ASC patients, age-matched cortical cataract patients and postmortem human lens (control). The mRNA level of Spry2 was determined using real-time PCR and normalized to GAPDH. ***P<0.001, NS: not significant, n = 6. Fold change relative to the level of the control groups is displayed. (**C**) Lens anterior capsule whole-mounts from ASC patients, age-matched cortical cataract patients, and postmortem human lens (control) were probed for Spry2 (green), α-SMA (red) and DAPI (blue). Images were acquired from the central area of each sample. Scale bar: 10μm.

It has been reported that Spry2 is expressed in whole lens during embryogenesis, and then becomes restrictively expressed in LECs[32]. Spatial regulation of Spry2 expression indicates its key regulatory role in LEC proliferation and differentiation in response to growth factors. Downregulation of Spry2 in mouse lens promoted TGFβ-induced EMT and formation of anterior subcapsular plaques[35]. In order to know whether Spry2 is deregulated in the development of ASC in human, we compared the expression level of Spry2 from anterior capsule LECs of ASC patients, cortical cataract patients and those from LECs of age-matched transparent lens (control). A representative slit-lamp microscope photo of an ASC patient showed a focal irregular opacity was formed beneath the anterior capsule (**Fig 1A**). Spry2 mRNA expression in LECs from ASC patients was decreased to approximately 1/3 of that from the human transparent lens, while the mRNA level of α-SMA was significantly increased (**Fig 1B**). Consistently, immunofluorescent staining of lens anterior capsule from ASC patients revealed decreased expression of Spry2 and increased expression level of α-SMA (**Fig 1C**). We also noticed that LECs from cortical cataract and ASC patients exhibited enlarged nuclei and abundant cytoplasm compared to those from transparent lens. However, Spry2 and α-SMA expression levels were changed only in LECs from ASC patients, but not in LECs from cortical cataract patients. The distinct expression patterns of Spry2 and α-SMA suggested that these two types of cataracts underwent different pathological changes. In ASC, LECs tend to transdifferentiate into myofibroblasts directly beneath the lens capsule, so a reduced expression level of Spry2 and a high level of EMT could be detected in these anterior capsule samples. Collectively, these data show that Spry2 is downregulated during human ASC formation, and negatively correlated with α-SMA upregulation.

### Spry2 negatively regulates TGFβ2-induced EMT in LECs

**Fig 2:**
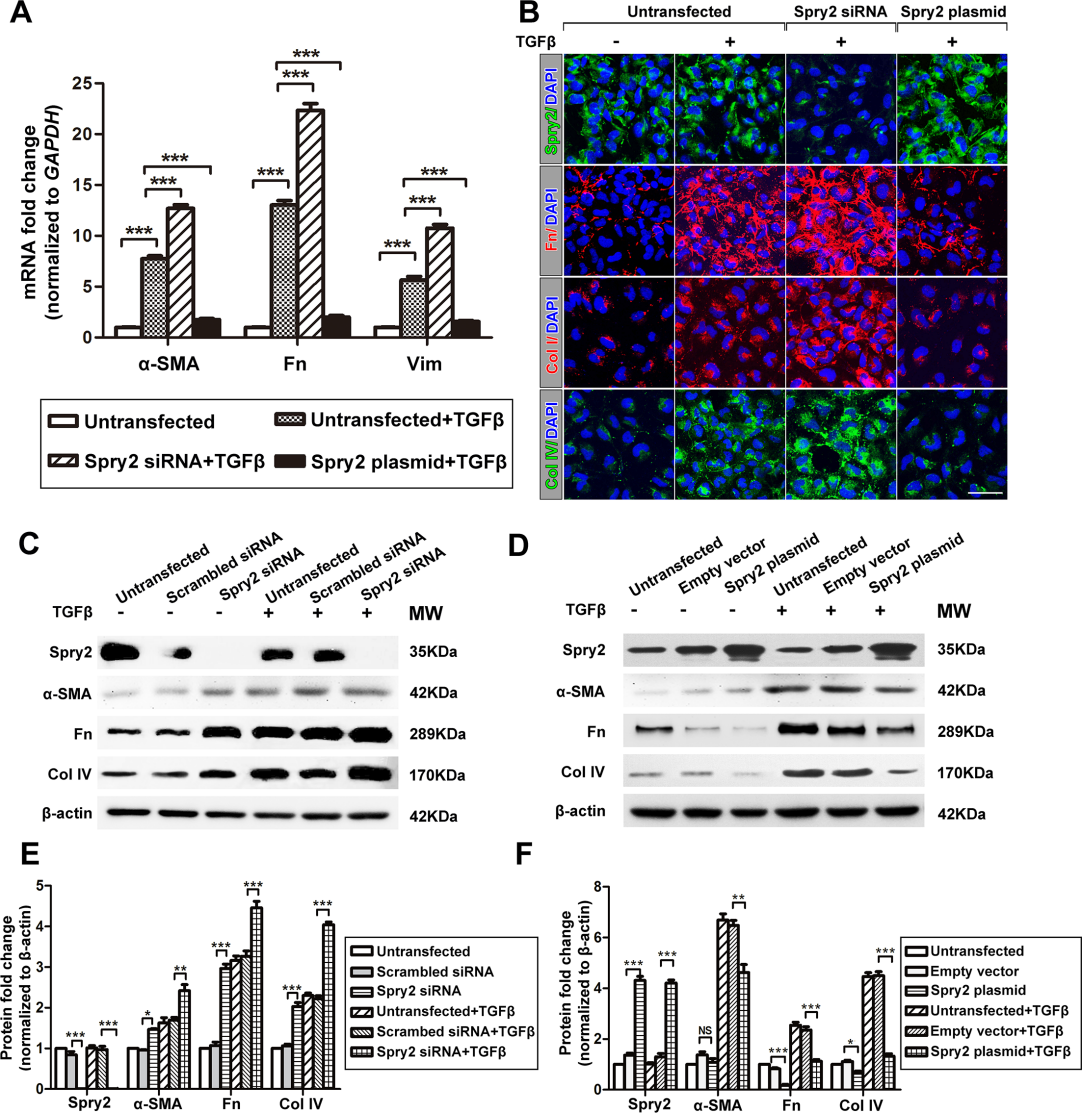
Spry2 inhibits TGFβ-induced EMT in human lens epithelial cells. **(A)** Cultured human lens epithelial cells were transfected with Spry2 siRNA or Spry2 plasmid and treated with or without TGFβ for 48h. The mRNA levels of α-SMA, Fn and Vim were determined by real-time PCR and normalized to GAPDH. Fold change relative to the level of the untransfected groups is displayed. **P<0.01, ***P<0.001, n = 3 **(B)** Cells were transfected with Spry2 siRNA or Spry2 plasmid and treated with or without TGFβ for 48h. Immunofluorescence was performed by probing Spry2, Fn, Col I, Col IV and DAPI. Scale bar: 50μm **(C-D)** Cells were transfected with Spry2 siRNA or Spry2 plasmid and treated with or without TGFβ for 48h. Untransfected cells, cells transfected with scrambled siRNA or empty vector were used as controls. Proteins were extracted and probed for Spry2, α-SMA, Fn, Col IV. β-actin was used as a loading control. **(E-F)** Quantification of the protein expression levels in C and D, respectively. Fold change relative to the level of the untransfected groups is displayed. *P<0.05, **P<0.01, ***P<0.001, NS: not significant, n = 3.

Since TGFβ2 plays a pivotal role in EMT and ASC development, it is likely that Spry2 downregulation will result in enhancement of TGFβ2-induced EMT. It has been reported in LECs that TGFβ2-induced EMT can be detected by upregulation of various mesenchymal cell markers, including α-SMA, Fn, Vim, Col I, and Col IV[12, 37]. Consistently, when we treated LECs by TGFβ2 for 48h, we found that the mRNA expression levels of α-SMA, Fn, and Vim were significantly increased (**Fig 2A**). Also, the protein expression levels of α-SMA, Fn, Col I, and Col IV were significantly increased (**Fig 2B–2F**), suggesting that LECs underwent EMT. To further determine the regulatory role of Spry2 in TGFβ2-induced EMT, we downregulated or overexpressed Spry2 in LECs during TGFβ2 treatment. When Spry2 was downregulated, the mRNA and protein expression levels of α-SMA, Fn, Col I, Col IV or Vim were significantly increased compared to those in LECs treated by TGFβ2 alone (**Fig 2A, 2B, 2C and 2E**). When Spry2 was overexpressed, the mRNA and protein expression levels of these EMT markers were significantly decreased compared to TGFβ2 treatment alone (**Fig 2A, 2B, 2D and 2F**). Collectively, these data indicate that Spry2 inhibits TGFβ2-induced EMT in LECs.

### Spry2 inhibits TGFβ2-induced LEC migration

**Fig 3:**
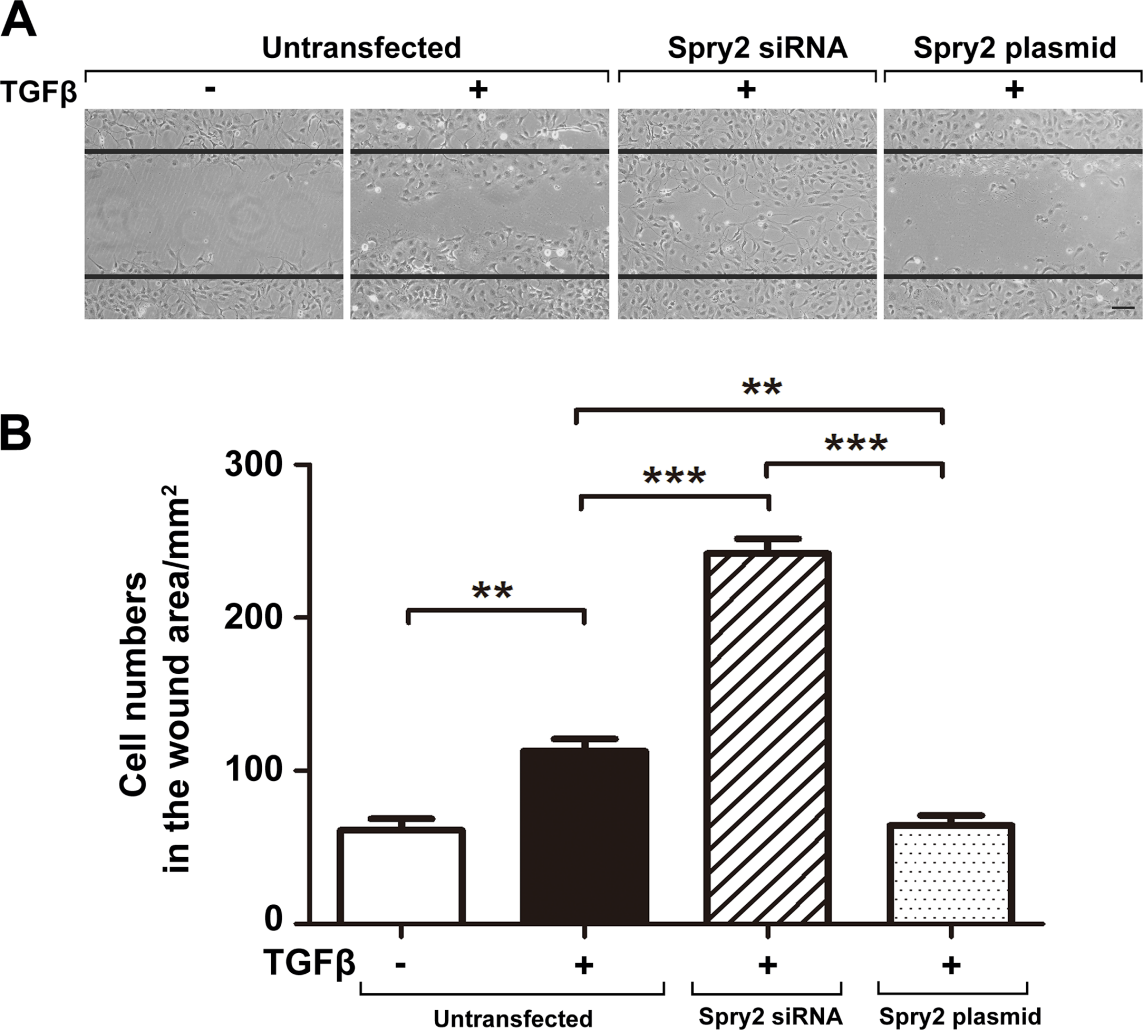
Spry2 prevents TGFβ-induced migration of human lens epithelial cells. **(A)** Cultured human lens epithelial cells were transfected with Spry2 siRNA or Spry2 plasmid and treated with or without TGFβ for 36h. Cell migration was observed by an inverted phase contrast microscope. Straight black lines indicate the wound edges. Scale bar: 100μm. **(B)** Quantification of the number of cells that migrated into the wound area. **P<0.01, ***P<0.001, n = 6.

EMT facilitates cell migration by degradation of the underlying basement membrane[38]. After cataract surgery, TGFβ in the aqueous humor facilities the migration of LECs to the posterior capsule, and blockade of TGFβ pathway inhibits LECs migration[12, 26]. To assess whether Spry2 also negatively regulates TGFβ2-induced LECs migration, we performed wound scratch assays. Consistent with previous studies[39, 40], the number of LECs in the wound area was significantly increased after treatment by TGFβ2 for 36 hours. Cell migration was enhanced upon Spry2 downregulation and suppressed upon Spry2 overexpression (**Fig 3A and 3B**). These findings demonstrate that Spry2 negatively regulates TGFβ2-induced LECs migration, which is a crucial stage in the pathogenesis of PCO.

### Spry2 suppresses TGFβ2 signaling through inhibition of Smad2 and ERK1/2 phosphorylation

**Fig 4:**
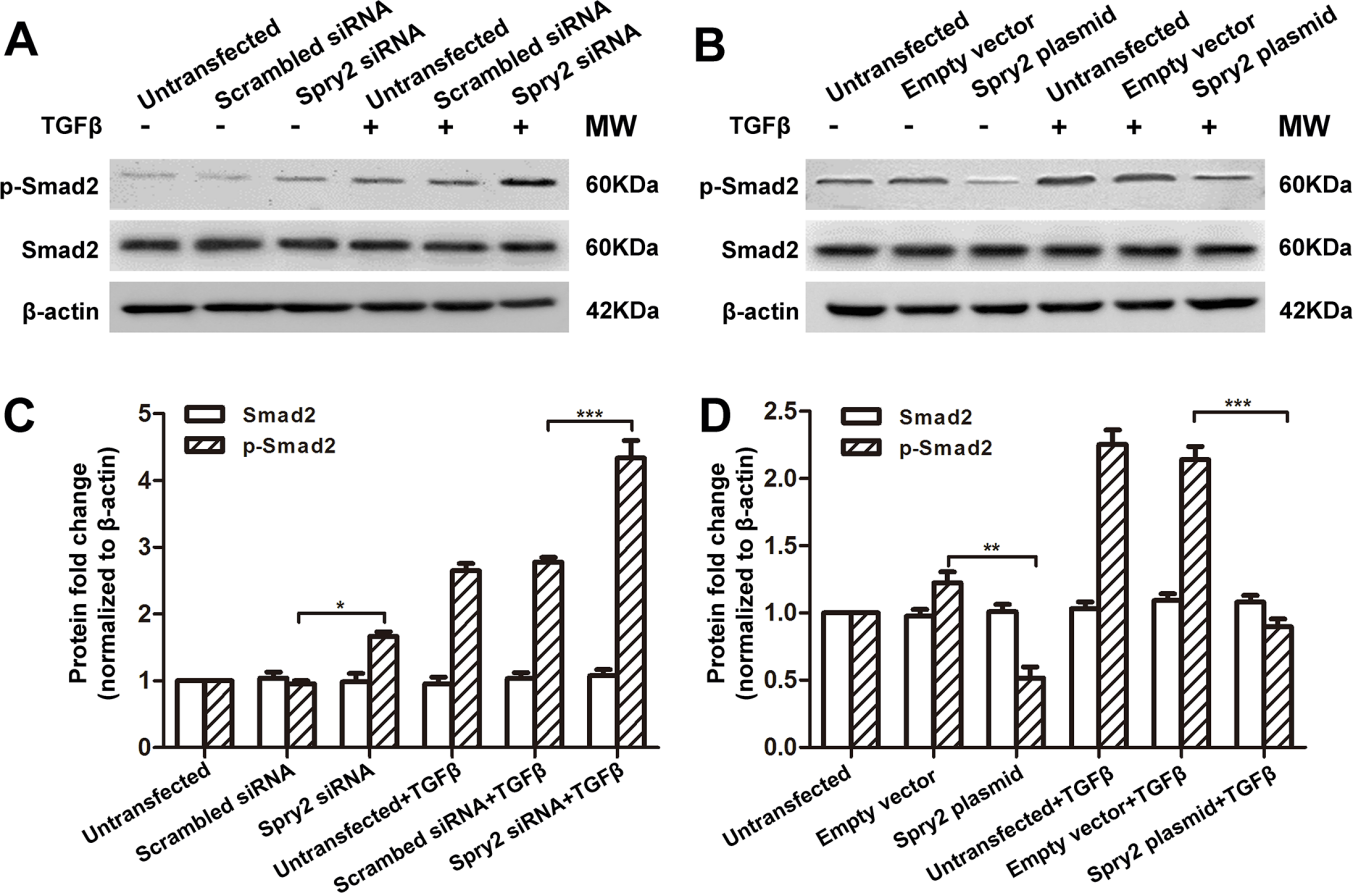
Spry2 suppresses Smad2 phosphorylation in human lens epithelial cells. **(A-B)** Cultured human lens epithelial cells were transfected with Spry2 siRNA or Spry2 plasmid and treated with or without TGFβ for 48h. Untransfected cells, cells transfected with scrambled siRNA or empty vector were used as controls. Proteins were extracted and probed for pSmad2 and total Smad. β-actin was used as a loading control. **(C-D)** Quantification of the pSmad2 and total Smad expression levels in A and B, respectively. Fold change relative to the level of untransfected groups is displayed. *P<0.05, **P<0.01, ***P<0.001, n = 3.

**Fig 5:**
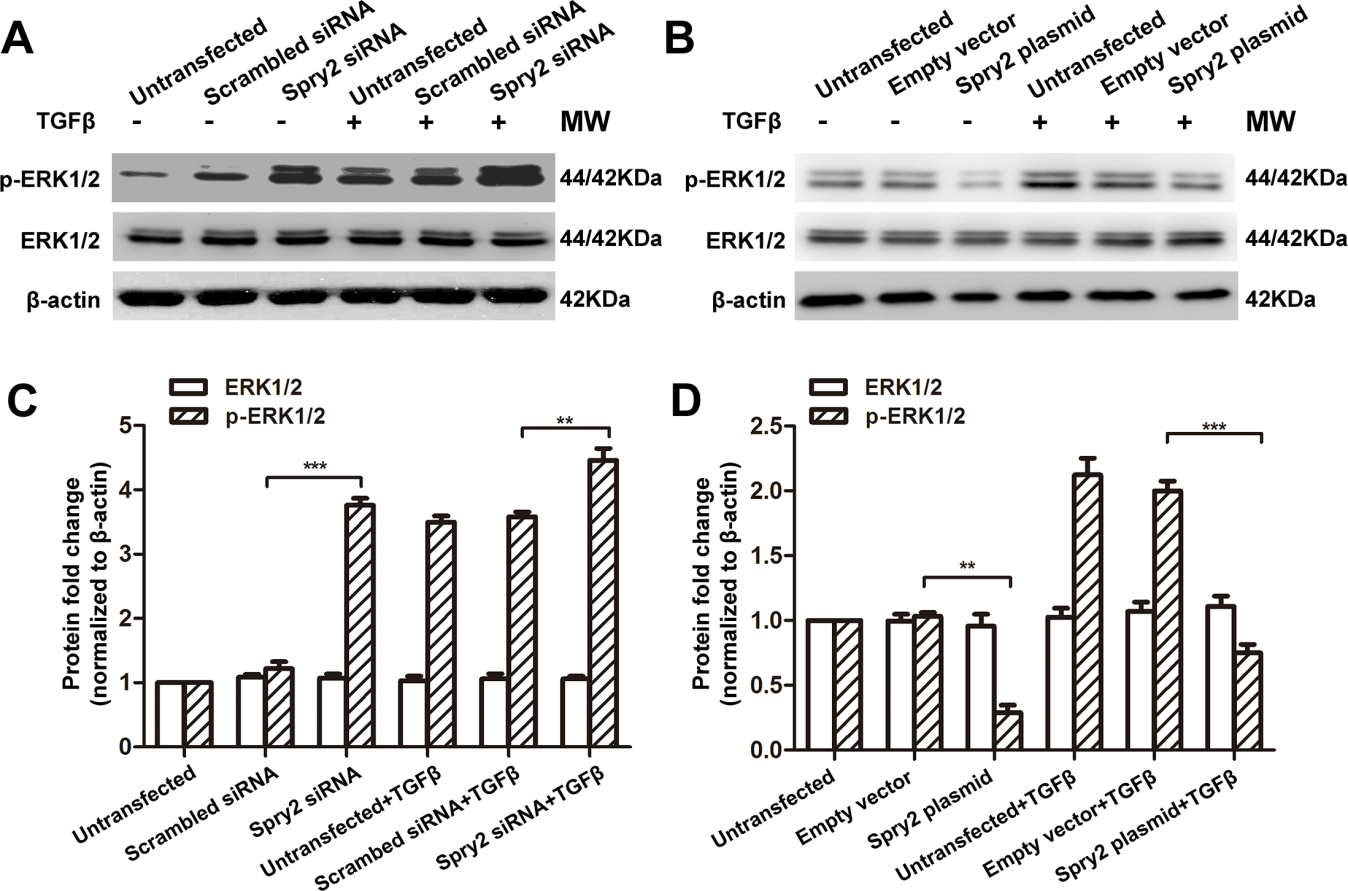
Spry2 suppresses ERK1/2 phosphorylation in human lens epithelial cells. **(A-B)** Cultured human lens epithelial cells were transfected with Spry2 siRNA or Spry2 plasmid and treated with or without TGFβ for 48h. Untransfected cells, cells transfected with scrambled siRNA or empty vector were used as controls. Proteins were extracted and probed for pERK1/2 and total ERK1/2. β-actin was used as a loading control. **(C-D)** Quantification of the pERK1/2 and total ERK1/2 expression levels in A and B, respectively. Fold change relative to the level of untransfected groups is displayed. **P<0.01, ***P<0.001, n = 3.

The negative regulatory role of Spry2 in of TGFβ2-induced EMT and migration suggest that Spry2 is an inhibitor of TGFβ signaling. To further dissect the mechanism of how Spry2 regulates TGFβ2-induced LECs EMT and migration, we measured the phosphorylation levels of Smad2 and ERK1/2, which were involved in the activation of canonical and non-canonical TGFβ signaling pathways, respectively[17, 18]. Consistent with our previous study[41], TGFβ treatment for 48 hours significantly increased the phosphorylation level of Smad2 and ERK1/2 in LECs, while the total expression level of Smad2 and ERK1/2 remained constant (**Figs 4 and 5**). Phosphorylation levels of Smad2 and ERK1/2 in LECs were significantly increased when Spry2 was downregulated, compared to those in LECs treated with TGFβ2 alone (**Figs 4A, 4C, 5A and 5C**). Also, phosphorylation levels of Smad2 and ERK1/2 levels were significantly decreased when Spry2 was overexpressed (**Figs 4B, 4D, 5B and 5D**). These data suggest that Spry2 is a potent negative regulator of TGFβ2 signaling through inhibition of both canonical and non-canonical TGFβ2 signaling pathways.

### Inhibition of Smad2 or ERK1/2 suppresses Spry2 KD-induced EMT

**Fig 6:**
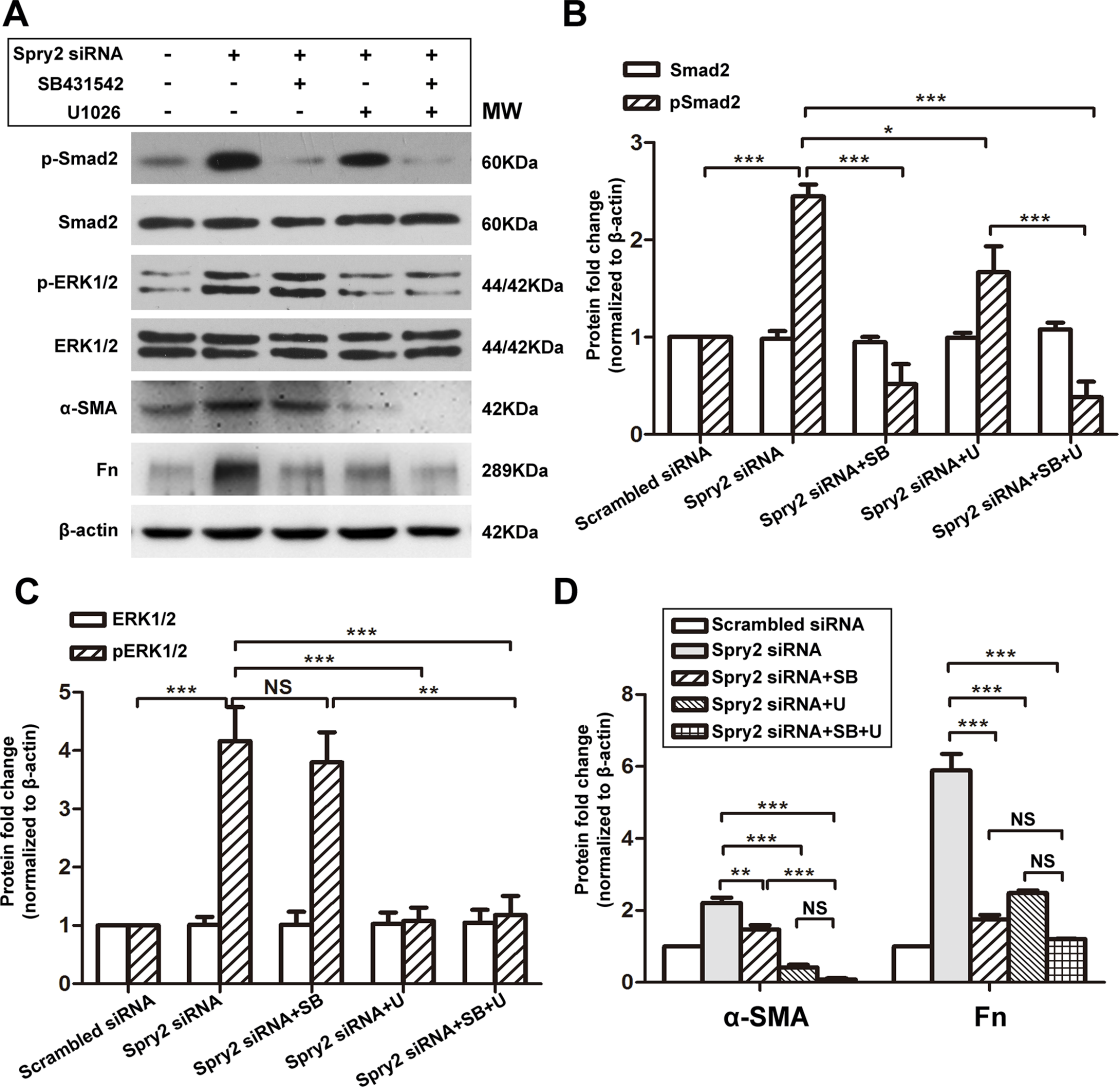
EMT induced by Spry2 downregulation was inhibited by Smad and ERK1/2 specific inhibitors. **(A)** Cultured human lens epithelial cells were transfected with scrambled siRNA or Spry2 siRNA and treated with Smad inhibitor SB431542 (10.0μM) or ERK1/2 inhibitor U1026 (10.0μM) or both for 24h. Proteins were extracted and probed for pSmad, total Smad, pERK1/2, total ERK1/2, α-SMA and Fn. β-actin was used as a loading control. **(B-D)** Quantification of the protein expression levels in A. Fold change relative to the level of the scrambled siRNA transfected group is displayed. *P<0.05, **P<0.01, ***P<0.001, NS: not significant, n = 3.

Interestingly, we noticed that even in conditions without TGFβ2 treatment, the expression levels EMT markers α-SMA, Fn, and Col IV were increased upon Spry2 downregulation and were decreased upon Spry2 overexpression (**Fig 2C–2F**). Also, without TGFβ2 treatment, there was a significant increase of p-Smad2 and p-ERK1/2 upon Spry2 downregulation and a significant decrease of p-Smad2 and p-ERK1/2 upon Spry2 overexpression (**Figs 4 and 5**). This can be explained by the basal activity of TGFβ and some other growth factors or cytokines that were contained in FBS[42] or secreted by LECs[43]. Also, these results suggested that Spry2 was a downstream regulator in TGFβ pathway. To further investigate how Spry2 inhibits TGFβ2 signaling, we treated Spry2 KD LECs with kinase inhibitors SB431542 and U0126, respectively. SB431542 inhibits TGFβ2 signaling by abrogating Smad2 phosphorylation and nuclear translocation[44]. U0126 is a specific inhibitor for ERK1/2 phosphorylation[45]. We found that SB431542 significantly suppressed upregulation of pSmad2 due to Spry2 downregulation, but did not affect pERK1/2 level. However, U1026 significantly suppressed upregulation of both pERK1/2 and pSmad2 due to Spry2 downregulation (**Fig 6A–6C**). These data confirmed our previous finding that canonical Smad signaling can be at least partially induced by noncanonical ERK1/2 signaling[41, 46]. We also found that SB431542 or U0126 treatment for 48 hours significantly suppressed upregulation of α-SMA and Fn due to Spry2 downregulation in LECs (**Fig 6A and 6D**). Moreover, we observed an enhanced suppression of α-SMA by combined treatment with both SB431542 and U1026 compared to treatment with SB431542 alone. Collectively, these results further supported that Spry2 suppressed TGFβ signaling by inhibiting Smad2 and ERK1/2 phosphorylation.

## Discussion

**Fig 7:**
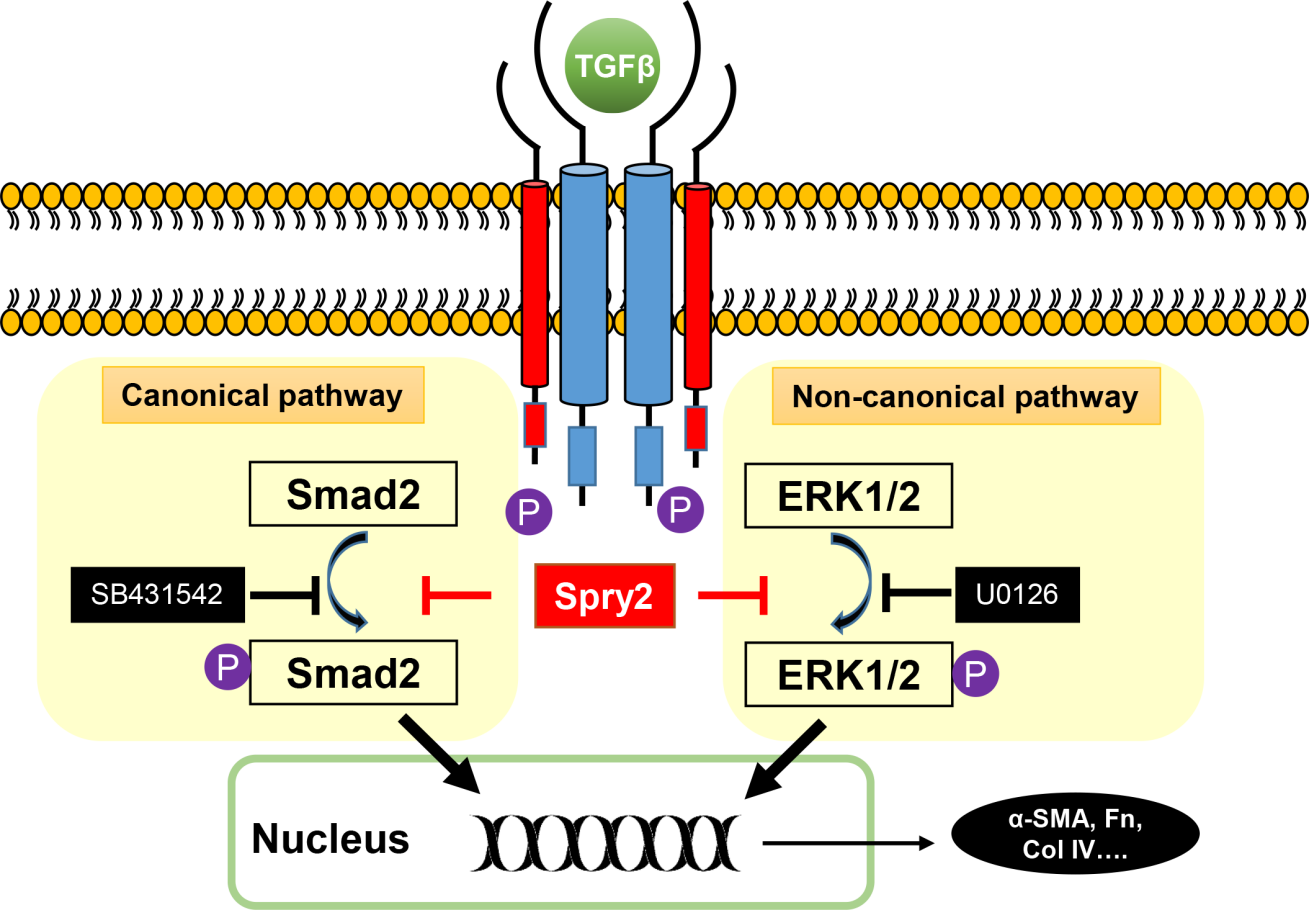
A working model for Spry2 in regulation of TGFβ signaling pathway. TGFβ signals through canonical pathway and non-canonical pathway. The canonical pathway needs phosphorylation of Smad2, and can be blocked by SB431542. The non-canonical pathway needs phosphorylation of ERK1/2, and can be inhibited by U0126. Spry2 can block both canonical and non-canonical pathways, thus suppress TGFβ-induced EMT.

Various growth factors, including fibroblast growth factor (FGF), hepatocyte growth factor (HGF), epidermal growth factor (EGF), and TGF, contribute to the development of ASC and capsule opacification[47, 48]. Inhibition of specific growth factor signaling pathways, such as TGF[12, 35] or EGF[49], could prevent formation of ASC or PCO. Ideally, one effective approach for ASC or PCO prevention is to target a common signal protein in multiple growth factor pathways. Growth factors mainly signal through activation of RTK. Intriguingly, Spry2 is a general inhibitor in RTK signaling, such as FGF, EGF and TGF[29, 35]. In this study, we demonstrated for the first time that Spry2 is expressed in human LECs, and that Spry2 is downregulated in the anterior capsule of ASC patients. Also, in human LECs, Spry2 negatively regulates TGFβ2-induced EMT and migration through inhibition of both Smad2 and ERK1/2 phosphorylation. These data combine to suggest a critical role of Spry2 in TGFβ2-induced EMT of LECs and human ASC formation (**Fig 7**). Although the regulatory role of Spry2 on other growth factor signaling pathways in LECs remains to be investigated, inhibition of ERK1/2 by Spry2 suggests that Spry2 may also negatively regulate FGF and EGF signaling in human LECs. Therefore, targeting Spry2 may be a potent strategy for controlling ASC and capsule opacification.

It has been reported that in mouse, conditional KD of Spry2 in lens elevates RTK-mediated ERK1/2 activation and facilitates TGF-induced EMT[35]. Also, overexpression of Spry2 in the mouse lens suppresses LECs proliferation and differentiation by inhibiting RTK signaling[34]. Following these studies, several questions need to be addressed. First, whether Spry2 is expressed in human lens and how it is regulated during human ASC and capsule opacification developments need to be characterized. Second, growth factor signaling pathways between mouse and human, such as FGF and TGF, are not completely identical[50, 51], and the expression profiles of crystalline and phospholipids between mouse and human lens are different[52]. These findings highlight that an evolutionary diversity may exist between species in the context of lens diseases. Moreover, recent findings suggest that Spry2 performs atypical roles in EMT in different cell types. Spry2 downregulation in colorectal epithelium inhibits EMT and serves as a biomarker for poor prognosis of colorectal cancer [53]. Conversely, Spry2 downregulation in breast epithelial cells induces EMT and migration[54]. Different responses to Spry2 alteration between different cell types may be caused by cell-type specific Spry targets[53, 55].Therefore, it is of great significance to characterize the regulatory role of Spry2 in human lens tissues and human LECs. Our study for the first time demonstrated the expression of Spry2 in human lens, and proved that it has an inhibitory role in TGFβ2-induced EMT in human LECs.

TGFβ signals through phosphorylation of Smad2/3 and ERK1/2. Although Spry family is known as an inhibitor of ERK1/2 phosphorylation, we demonstrated in human LECs that Spry2 can also inhibit activation of Smad2, which is consistent as previously reported in mouse[35]. Spry2 contains a canonical Casitas B-lineage lymphoma tyrosine kinase-binding motif(c-Cbl TKB): N-X-Y(p)-S/T-X-X-P with a key tyrosine residue (Y55)[56]. Binding to the c-Cbl TKB to protein phosphatase 2A(PP2A) exposes its C-terminal cryptic SH3-binding motif, and promotes the interactions between Spry2 with adaptor protein GRB2 and other SH3-containing targets, thus leading to inhibition of the downstream effector ERK1/2[57]. Since current data and our previous findings all suggest that Smad2 phosphorylation can be at least partially induced by ERK1/2 phosphorylation[41, 46], and ERK can stabilize pSmad2[58], it is likely that downregulation of pSmad2 by Spry2 overexpression is a consequence of ERK1/2 inhibition. Whether Spry2 can directly interact with Smad2 needs further investigation. But according to our data, combined treatment with SB431542 and U1026 exerted more suppression on α-SMA compared to SB431542 treatment alone, suggesting that EMT caused by Smad2 and ERK1/2 activation are not fully in one tandem pathway, and that Spry2 may have a negative regulatory role on both.

Phosphorylation of Smad2 can occur at Ser 465/467 or Ser 245/250/255. Phosphorylation of Smad2 at Ser 465/467 by the TGFß receptor positively regulates TGFß signaling, while phosphorylation of Smad2 at Ser 245/250/255 by MAP kinase negatively regulates TGFß signaling[59]. In this study, we demonstrated that Spry2 suppressed TGFß-induced Smad2 phosphorylation at Ser 465/467. However, upon TGFβ2 treatment, phosphorylation of Smad2 can also occur at Ser 245/250/255 in lens epithelial cells[60]. Whether Spry2 also affects Smad2 phosphorylation at Ser 245/250/255 remains to be investigated.

Spry2 inhibits TGFβ signaling, but the expression of Spry2 can also be regulated by TGFβ through inhibition of Spry2 mRNA transcription or promoting Spry2 protein degradation[61]. Also, repressed expression of Spry2 can be caused by downregulation of miR-181a in LECs from PCO patients[62, 63]. Although we did not observe a downregulation of Spry2 in the presence of TGFβ in cultured human LECs, we found that Spry2 was significantly downregulated in anterior capsule samples from ASC patients. This result suggested that the downregulation of Spry2 may be a trigger in the development of ASC and capsule opacification. Taken together, our work facilitates the understanding of fundamental biology by defining the role of Spry2 in human LECs transdifferentiation, and provides clinical evidence from human patients for the prevention of ASC and PCO development by using Spry2 as a potential therapeutic target.
